# Overexpression of MALAT1 Relates to Lung Injury through Sponging miR-425 and Promoting Cell Apoptosis during ARDS

**DOI:** 10.1155/2019/1871394

**Published:** 2019-12-01

**Authors:** Lu Wang, Jiao Liu, Wenjie Xie, Guang Li, Lan Yao, Rui Zhang, Bin Xu

**Affiliations:** ^1^Department of Critical Care Medicine, Renmin Hospital of Wuhan University, Wuhan, China; ^2^Department of Critical Care Medicine, School of Medicine, North Ruijin Hospital, Shanghai Jiaotong University, Shanghai, China; ^3^Department of Oncology, Renmin Hospital of Wuhan Unversity, Wuhan, China

## Abstract

**Background:**

Acute respiratory distress syndrome (ARDS) is a severe form of acute lung injury during which severe inflammatory responses induce cell apoptosis, necrosis, and fibrosis. Metastasis-associated lung adenocarcinoma transcript 1 (MALAT1) is a multiple function long noncoding RNA that was found overexpressed during acute lung injury. However, the roles of MALAT1 in ARDS patients are still unknown.

**Methods:**

Total RNA was extracted from the plasma, plasma exosome, and peripheral blood mononuclear cells (PBMCs) from 65 ARDS patients and 36 healthy controls. The MALAT1 and six candidate miRNAs levels were detected by qRT-PCR. The interaction between MALAT1 and miR-425 was predicted using a bioinformatics tool and verified by dual luciferase assay. Exosomes from ARDS patients were cultured with A549 and HFL-1 cells to confirm the delivery of miR-425 by exosomes. Cell apoptosis and viability were determined by flow cytometry and MTT assay.

**Results:**

We found MALAT1 was significantly increased in the ARDS patients' plasma and PBMCs. The MALAT1 level in PBMCs was negatively correlated with exosomal miR-425 level. MALAT1 interacted with miR-425 and protected phosphatase and tensin homolog (PTEN) expression in A549 and HFL-1 cells. Exosomes from ARDS patients delivered less miR-425 into A549 and HFL-1 cells and induced cell apoptosis via upregulating PTEN.

**Conclusion:**

This study identified increased MALAT1 and decreased miR-425 in ARDS patients and unveiled their roles during the pathogenesis of ARDS.

## 1. Introduction

Acute respiratory distress syndrome (ARDS) is a severe form of acute lung injury that occurs in critically ill or wounded patients which is characterized by widespread inflammation in the lungs and reduced oxygen uptake [1, 2]. During ARDS processes, severe inflammatory responses induce cell apoptosis, necrosis, and fibrotic agents releasing, which finally contribute to the pathogenesis of the lungs [3]. Mortality rate for patients with ARDS is very high, and many survivors suffered from complications such as breathing problems [4, 5]. Prediction of outcome in patients with ARDS is of major importance for appropriate treatment decisions and resource allocation. However, the complex etiology leads to complicated ARDS diagnosis and treatment. Although many protein-based biomarkers have been identified from patients with ARDS, none of them have been translated for ARDS clinical diagnosis [6].

Metastasis-associated lung adenocarcinoma transcript 1 (MALAT1) is a more than 8000 nt long nonprotein coding RNA (lncRNA), which is highly conserved among mammals [7, 8]. MALAT1 was first identified related to the poor prognosis of patients with non-small-cell lung adenocarcinoma [9]. Subsequently, increasing evidences indicated that MALAT1 is an important multiple function gene expression regulator, which not only contributes to the progression of tumors but also relates to maintaining normal physiological conditions [10], the aging processes [11], and the immune response [12, 13]. In an LPS-induced acute lung injury rat model, researchers found that MALAT1 knockdown plays protective roles by upregulating miR-146a [14]. However, the roles of MALA1 in ARDS are still unknown.

Exosomes are small extracellular vesicles derived from endosomal compartment vesicles budding from the plasma membrane [15]. Importantly, exosomes can be produced by almost all types of cells in culture and in various human body fluids including blood, saliva, urine, and breast milk [16]. As an important part of cell-cell communication, exosomes protect molecules from degradation and deliver specific functional proteins and RNAs from supplier cells to receiver cells [17]. Recently, researchers found that exosomes derived from endothelial progenitor cells ameliorate acute lung injury by transferring miR-126 to target endothelial cells [18]. MALAT1, as a nuclear localized lncRNA, has also been found to be degraded into segments, packaged into exosomes, and finally transferred into target cells [19, 20].

Phosphatase and tensin homolog (PTEN) is a tumor suppressor which can modulate the PI3K pathway by catalyzing degradation of PI3K-generated PIP3 [21]. In this manner, PTEN restrains cell proliferation through inhibiting downstream functions of the PI3K-Akt pathway. PTEN is robustly expressed in normal lung fibroblasts, and the downregulation of PTEN is related to aberrant fibroblast proliferation and collagen secretion during LPS-induced acute lung injury [22–24].

In the present study, we examined the MALAT1 and 6 candidate miRNAs levels in plasma, plasma exosome, and peripheral blood mononuclear cells (PBMCs) from 65 ARDS patients and 36 healthy controls. We analyzed the correlation between MALAT1 and miRNAs. Exosomes coculture with lung fibroblasts, and alveolar epithelial cells were employed to analyze the exosome-delivered MALAT1 function.

## 2. Materials and Methods

### 2.1. Study Population

65 ARDS patients and 36 healthy volunteers were obtained from Renmin Hospital of Wuhan University hospital. The clinical characters are listed in Table 1. All ARDS subjects met the Berlin diagnostic definition [5]: timing of ARDS was within 1 week of a known clinical insult or new or worsening respiratory symptoms; chest imaging showed bilateral opacities (not fully explained by effusions, lobar/lung collapse, or nodules); respiratory failure was not fully explained by cardiac failure or fluid overload; and ARDS severity was based on PaO_2_/FiO_2_ ratio (including 31 moderate ARDS patients and 34 severe ARDS patients). Patients with diffused alveolar hemorrhage or chronic lung disease were excluded. Treatment with the granulocyte colony-stimulating factor or inhibitors of tumor necrosis factor was also excluded [25]. Patients were enrolled in the study immediately after meeting all inclusion criteria [5]. Institutional review boards of Renmin Hospital of Wuhan University hospital approved this study. Five milliliters of blood samples were collected within the first 24 h. Half of the samples were immediately centrifuged at 3000 ×g for 15 min at 4°C. Plasma was removed and stored at − 80°C until further assessment. Another half was subjected to cell separation using Ficoll Histopaque (Sigma).

Controls were free of lung, cardiac, infectious, and allergic diseases and without any prescribed chronic diseases.

### 2.2. Exosome Isolation from Plasma Samples

Exosomes were extracted from plasma using ExoQuick Exosome Precipitation Solution (System Biosciences, Mountain View, Calif). Briefly, plasma was obtained by centrifugation at 3000 ×g for 15 minutes to remove cells and cellular fragments, and subsequent filtration of the supernatant was accomplished through a 0.45-*μ*m pore polyvinylidene fluoride filter (Millipore, Billerica, Mass). ExoQuick was added to the supernatants, and exosomes were precipitated by refrigeration at −20°C for 12 hours. Exosome pellets collected by centrifugation at ×1500 g for 30 minutes were dissolved in 20 *μ*L PBS.

### 2.3. RNA Extraction

Total RNA was extracted from cells, plasma, and exosomes using Trizol reagent (Invitrogen, Carlsbad, CA, USA) according to the manufacturer's instructions. cel-miR-39 was added to plasma and EV samples as an external reference. RNA concentration and purity were determined using a model ND-2000 spectrophotometer (Nanodrop Technologies, Wilmington, DE, USA). Only samples with absorbance ratios 260 nm/280 nm of ∼2.0 and 260 nm/230 nm of 1.9–2.2 were considered for inclusion in the study.

### 2.4. Quantitative RT-PCR

Quantitative RT-PCR analysis was used to determine the relative level of selected MALAT1 and miRNAs. Primers for MALAT1 quantification were: F-GAATTGCGTCATTTAAAGCCTAGTT, R-GTTTCATCCTACCACTCCCAATTAAT; GAPDH: F-ACAGTCAGCCGCATCTTCTT, R-GACAAGCTTC CCGTTCTCAG. The levels of miRNAs were detected by TaqMan miRNA RT-Real Time PCR. Single-stranded cDNA was synthesized using TaqMan MicroRNA Reverse Transcription Kit (Applied Biosystems, Foster City, CA, USA) and then subjected to qPCR using miRNA-specific TaqMan MGB probes (Applied Biosystems). Cel-miR-39 was used for normalization in plasma and EV samples. U6 snRNA was used for normalization in cells. Each sample in each group was measured in triplicate, and the experiment was repeated at least three times. The relative MALAT1 and miRNAs levels were compared using the 2-ΔΔCq method.

### 2.5. Plasmids Construction

Full length of 8708 bp MALAT1 was cloned into pcDNA3.1 vector between Xho I and Not I restriction sites. To generate MALAT1 luciferase reporter, a segment of 3325 bp of MALAT1, containing predicted miR-425 binding site was cloned downstream of the firefly luciferase gene in the pmirGLO plasmid (Promega, Madison, WI, USA). To generate PTEN reporter, a segment of 476 bp segment containing miR-425 binding site was cloned into pmirGLO vector downstream of the firefly luciferase coding region.

### 2.6. Cell Culture

Human lung fibroblasts HFL-1 were obtained from the American Type Culture Collection (ATCC, Manassas, VA, USA). Human alveolar epithelial A549 cells were purchased from the Chinese National Infrastructure of Cell line Resources. All these cells were cultured in Dulbecco's Modified Eagle Medium containing 10% fetal bovine serum (Hyclone, Logan, UT, USA), 100 IU/ml penicillin, and 100 IU/ml streptomycin. All cells were maintained at 37°C under an atmosphere of 5% CO_2_.

### 2.7. Dual Luciferase Assay

miR-425 mimic was purchased from GenePharma Co., Ltd. (Shanghai, China). For the luciferase reporter assay, HFL-1 and A549 cells were seeded in 48-well plates. Luciferase reporter vectors were cotransfected with miR-425 mimic using lipofectamine 2000 (Invitrogen). 48 hours after transfection, cells were lysed, and the cell lysates were subjected to dual luciferase assay with the Dual-Luciferase Assay kit (Promega, Madison, WI, USA). Each treatment was performed in triplicate in three independent experiments. The results are expressed as relative luciferase activity (firefly luciferase/renilla luciferase).

### 2.8. Immunoblotting

Proteins were extracted from cell samples and then separated by electrophoresis after boiling in sodium dodecyl sulfate/*β*-mercaptoethanol sample buffer. The proteins in the gels were blotted onto a polyvinylidene fluoride membrane (Amersham Pharmacia Biotech, St. Albans, Herts, UK) by electrophoretic transfer. After blocking by 5% nonfat milk for 1 hour at room temperature, the membrane was incubated with rabbit anti-PTEN polyclonal antibody (Abcam, Cambridge, MA, USA) or mouse anti-*β*-actin monoclonal antibody (Santa Cruz Biotechnology Inc., Santa Cruz, CA, USA) overnight at 4°C. After washing three times, the membranes were incubated with horseradish peroxidase-conjugated goat anti-rabbit or rabbit anti-mouse secondary antibody for another 2 hours at room temperature. Detection by the chemiluminescence reaction was carried out using ECL kit (Pierce, Appleton, WI, USA). The *β*-actin signal was used as a loading control.

### 2.9. Cell Proliferation Assay

Cell proliferation was estimated by the 3-(4,5-dimethylthiazol-2-yl)-2,5-diphenyltetrazolium bromide (MTT) assay. A549 and HFL-1 cells were seeded in wells of 96-well plates at low density (2 × 10^3^) in DMEM medium and allowed to attach overnight. The cells were then cocultured with exosomes from ARDS patients or healthy controls. Twenty microliters of MTT (5 mg/mL) (Sigma-Aldrich) were added to each well 48 h after coculture, and the cells were incubated for further 4 h. The absorbance was recorded at 570 nm with a 96-well plate reader after addition of dimethyl sulfoxide (DMSO).

### 2.10. Flow Cytometry

A549 and HFL-1 cells were seeded in wells of 12-well plates at low density (3 × 10^5^) in DMEM medium and allowed to attach overnight. The cells were cocultured with exosomes from ARDS patients or healthy controls for 48 hours and then subjected to flow cytometry after staining by FITC-Annexin V and propidium iodide. We followed the methods of Li et al. [26].

### 2.11. Statistical Analysis

Data were analyzed using SPSS Statistical Package version 19.0 (SPSS Inc., Chicago, IL, USA). Two-tailed Student's *t*-test was used to calculate statistical significance between two comparator groups. The correlation analysis was analyzed by χ^2^-analysis. A two-tailed *P* value < 0.05 was considered significant.

## 3. Results

**Figure 1:**
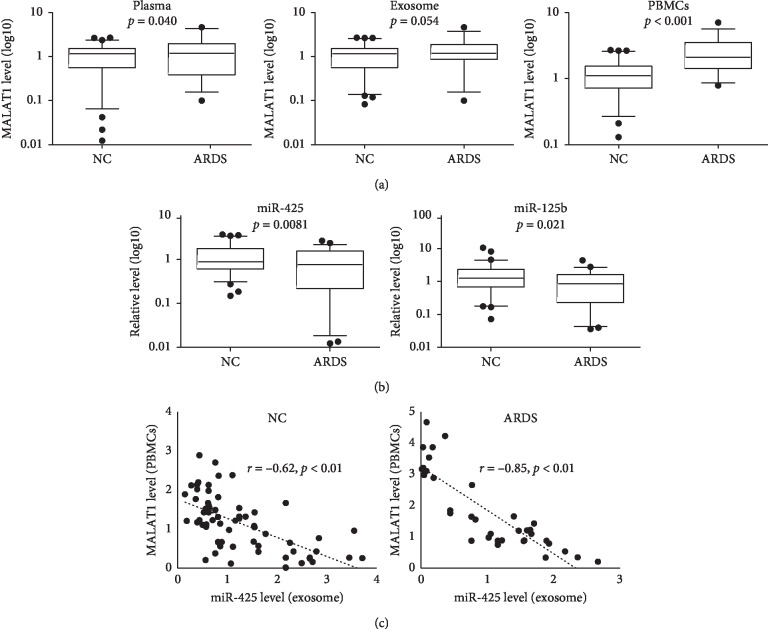
MALAT1 level increased in ARDS patients' plasma and PBMCs, and negatively correlates with miR-425 level in exosomes. (a) Total RNAs were extracted from plasma, plasmal exosomes, and PBMCs from ARDS patients and healthy controls, and the MALAT1 level was quantified by qRT-PCR. (b) Total RNAs were extracted from plasmal exosome and candidate miRNA level was detected by qRT-PCR. Results were shown as box-whisker diagram and data out of 95% CI were shown as dots. (c) Correlation between PBMCs MALAT1 level and exosomal miR-425 level.

To understand the roles of MALAT1 in ARDS, the MALAT1 level in plasma, PBMCs, and plasma exosomes was quantified by RT-PCR. We found the MALAT1 level was significantly increased in the plasma and PBMCs from ARDS patients (Figure 1(a)). It is known that some miRNAs were disturbed when ARDS occur; thus, we detected 6 candidate miRNAs in the exosome (Figure 1(b) and [Supplementary-material supplementary-material-1]). We chose these miRNAs because these miRNAs' level altered in the ARDS patients or mouse models [27–31]. We found miR-425 and miR-125b were significantly reduced in the patients with ARDS (Figure 1(b)). It is reported that MALAT1 can function as a miRNA sponge, protecting gene expression through interaction with miRNAs [32, 33]. So, we hypothesize that MALAT1 level in the cells may relate to the miRNAs level in the extracellular fluids. After correlation analysis, we found MALAT1 level in the PBMCs was negatively correlated with plasma miR-425 in ARDS patients and healthy controls (Figure 1(c)).

**Figure 2:**
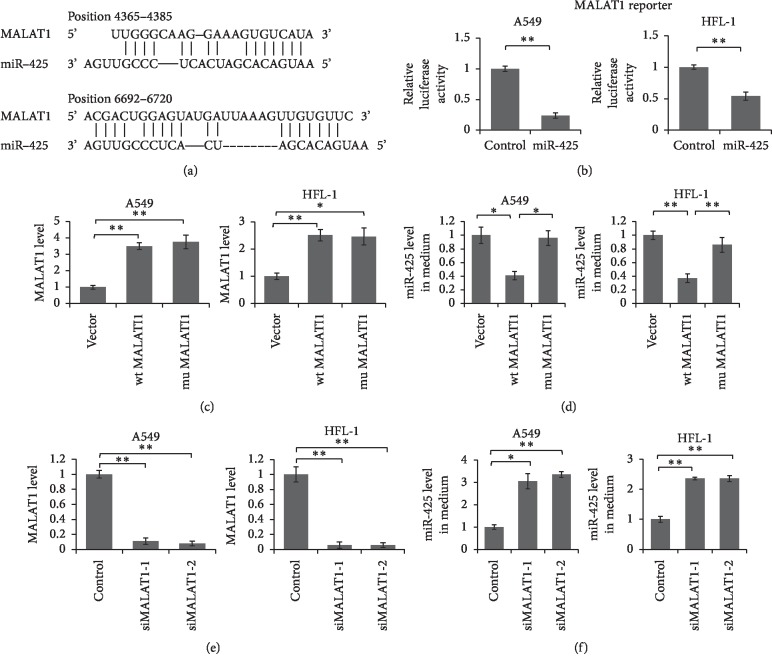
MALAT1 directly interacting with miR-425 and up-regulated PTEN expression through sponging miR-425. (a) Schematic diagram of predicted interaction between MALAT1 and miR-425. (b) Dual luciferase assay. A549 and HFL-1 cells were transfected with MALAT1 reporter vector and miR-425 mimic or control oligo for 48 hours. Cells were lysed and subjected to luciferase assay. Results were analyzed by Student's *t*-test, and *p* < 0.05 was considered significant. (c) A549 and HFL-1 cells were transfected with wildtype or mutant MALAT1 expression vector, with empty vector as control. The MALAT1 level was detected 48 hours after transfection. MiR-425 level in the cell culture medium was detected by qRT-PCR at the same time (d). (e) A549 and HFL-1 cells were transfected with MALAT1 specific siRNAs for 48 hours. The MALAT1 level in A549 and HFL-1 was detected by qRT-PCR. (f) The miR-425 level in the medium was quantified by qRT-PCR. Results were analyzed by *t*-test, and *p* < 0.05 was considered significant. ^*∗*^*p* < 0.05, ^*∗∗*^*P* < 0.01.

Subsequently, we predicted the interaction between MALAT1 and miR-425 using bioinformatics tool: RNAhybrid ([https://bibiserv2.cebitec.uni]). We found there are at least two regions in the MALAT1 which have the potential to bind with the miR-425 seed sequence (Figure 2(a)). To confirm the interaction between MALAT1 and miR-425, we cloned a segment of 3325 bp of MALAT1 into pmirGLO vector, following firefly luciferase coding region to obtain the reporter vector. HFL-1 and A549 cells were transfected with MALAT1 reporter vector and miR-425 mimic for 48 hours. Cells were lysed for dual luciferase assay. As shown in Figure 2(b), the relative luciferase activities were significantly repressed by miR-425 mimic when compared with control oligo.

To further unveil the correlation between cellular MALAT1 and exosomal miR-425, we constructed wildtype and miR-425 interaction sites deleted mutant MALAT1 expression vector. We overexpressed wildtype or mutant MALAT1 in A549 and HFL-1 cells, and the level of miR-425 in cells and medium was detected by qRT-PCR. As shown in Figure 2(c), the MALAT1 level was successfully overexpressed up to more than twofold in A549 and HFL-1 cells. Meanwhile, the miR-425 levels in the medium were significantly reduced when wildtype MALAT1 was overexpressed in A549 and HFL-1 cells (Figure 2(d)). However, no significant change of miR-425 level in the cells was found in these two cell lines ([Supplementary-material supplementary-material-1]). Meanwhile, when MALAT1 was knocked down by siRNA (Fig. 2E), the miR-425 level in the medium was significantly increased (Fig. 2F). These results indicated that MALAT1 inhibited miR-425 secretion out of the cells but did not repress miR-425 expression in the cells.

**Figure 3:**
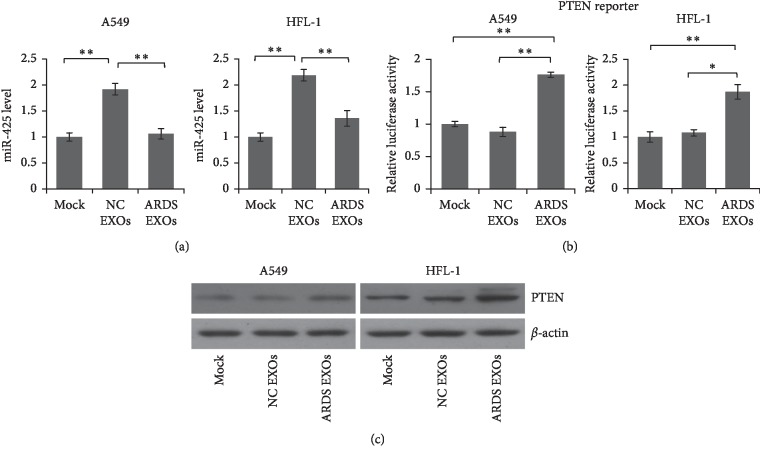
Exosome from ARDS patients' plasma delivered less miR-425 into target cells and did not repress PTEN expression. (a) A549 and HFL-1 cells were cocultured with exosomes from ARDS patients or healthy controls. The miR-425 level was detected by qRT-PCR 48 hours after coculture. (b) A549 and HFL-1 cells were transfected with PTEN reporter and then cocultured with exosomes from ARDS patients or healthy controls. The luciferase activity was detected 48 hours after coculture. Results were analyzed by *t*-test, and *p* < 0.05 was considered significant. ^*∗*^*p* < 0.05, ^*∗∗*^*P* < 0.01. (c) A549 and HFL-1 cells were cocultured with exosomes from ARDS patients or healthy controls. The PTEN protein level was determined by immunoblotting.

To explore the function of the exosomes from ARDS patients, A549 and HFL-1 cells were cocultured with exosomes from ARDS patients and healthy controls for 72 hours. As shown in Figure 3(a), the miR-425 level was significantly decreased in the cells cocultured with ARDS patients' exosomes. Meanwhile, the exosomes from ARDS patients can increase the luciferase activity in the cells transfected with PTEN reporter vector (Figure 3(b)). Immunoblotting was employed to detect the endogenous PTEN protein level in the cells after coculture, and the results showed that PTEN was increased in the cells cocultured with ARDS patients' exosomes (Figure 3(c)). These results indicated that exosomal miR-425 is functional and can be delivered into receiver cells.

**Figure 4:**
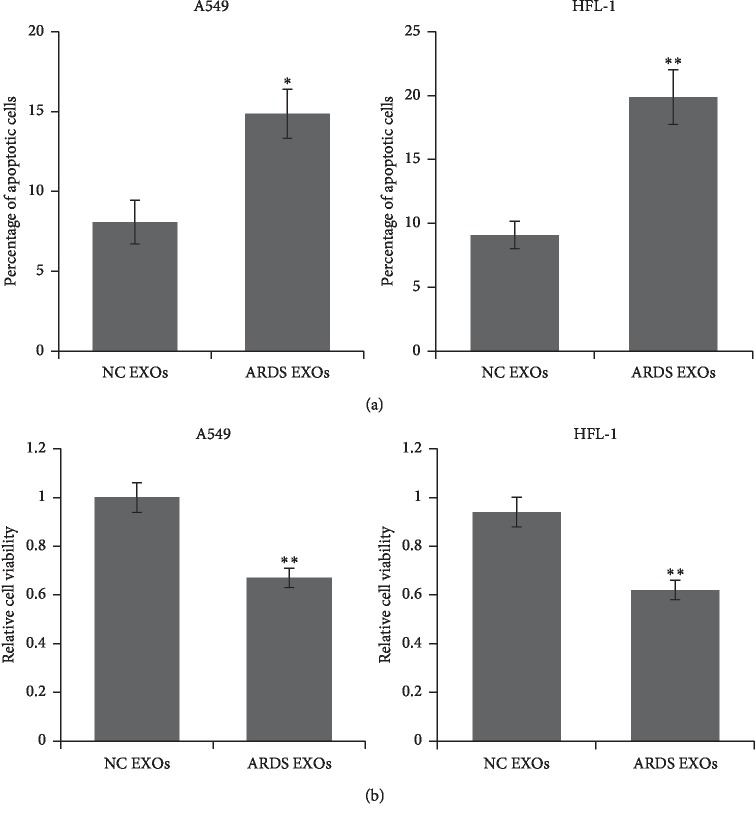
Exosomes from ARDS patients promoted cell apoptosis and inhibit cell proliferation. A549 and HFL-1 cells were cocultured with exosomes from ARDS patients or healthy controls. Apoptotic cells were detected by flow cytometry after FITC-Annexin V and PI staining (a). Cell viability was determined by MTT assay (b). Results were analyzed by Student's *t*-test, and *p* < 0.05 was considered significant. ^*∗*^*p* < 0.05 and ^*∗∗*^*P* < 0.01.

PTEN is an important tumor suppressor and induces cell apoptosis, so we detected the cell apoptosis and viability using A549 and HFL-1 cells after coculturing with exosomes. We observed increased apoptotic cell number (Figure 4(a)) and reduced cell viability (Figure 4(b)) in the cells cocultured with ARDS patients' exosomes.

## 4. Discussion

Acute respiratory distress syndrome (ARDS) is a respiratory failure which is associated with severe inflammatory reactions, interstitial and alveolar edema, cell infiltration into the alveolar space, and endothelial damage [34]. MALAT1 was found upregulated and relates to activation of nuclear factor kappa-light-chain-enhancer of activated B cells (NF-*κ*B) signaling in the lipopolysaccharide (LPS)-induced acute lung injury model [35]. MALAT1 was first identified as a pre-mRNA splicing regulator and mainly located in the cell nucleus [36]. Subsequently, MALAT1 was also found related to DNA double-strand break repair pathway [7]. Although MALAT1 is a nucleus-localized molecule, it has been found to be packaged into exosomes and delivered into the microenvironment and target cells [37, 38]. In this present study, we detected MALAT1 level in the plasma, plasmal exosomes, and PBMC from ARDS patients and want to explore the roles of MALAT1 during the pathogenesis of ARDS. We found the MALAT1 level was extremely high in the PBMCs from ARDS patients and negatively correlates with exosomal miR-425 level.

It is reported that miR-425 can promote cell proliferation in gastric cancer and contribute to the invasion and metastasis in hepatocellular carcinoma by targeting PTEN [39, 40]. However, the function of miR-425 in ARDS is still unknown. Herein, we confirmed that miR-425 can also repress PTEN expression in A549 and HFL-1 cells. Using dual luciferase assay and immunoblotting, we confirmed that MALAT1 can “sponge” miR-425 in the cells, protect PTEN expression, and inhibit miR-425 secretion into the medium. Thus, these evidences indicated that exosome-delivered miR-425 is an important cell apoptosis inhibitor in the lung microenvironment, and we hypothesized that overexpressed MALAT1 in ARDS patients may promote cell apoptosis and contribute to the lung pathophysiological process by sponging miR-425.

It is reported that PTEN can be packaged into exosomes and delivered into recipient cells [41, 42]. In the exosome coculture experiment, we observed increased miR-425 level in the NC exosome cocultured A549 and HFL-1 cells. However, as the target of miR-425, the PTEN protein level was similar in these two groups which indicated that exosome-delivered PTEN protein may increase the PTEN level in exosome cocultured cells.

Through coculture experiments, we confirmed that ARDS patients' exosomes delivered less miR-425 to the target cells, and induced cell apoptosis by upregulating PTEN expression. These results unveiled the biological role of increased MALAT1 in the cells and decreased miR-425 in the exosomes during ARDS. However, there are still some defects about this study. We found exosomal miR-425 level was reduced, and these exosomes delivered less miR-425, but we still do not know where these exosomes are from. These exosomes may come from the MALAT1 overexpressed PBMCs, which can explain the increased exosomal miR-425 by sponging theory. But the plasma exosomes are a mixture, so the MALAT1 level in the pathological tissues needs to be examined.

In conclusion, this study identified two dysregulated noncoding RNAs (MALAT1 and miR-425) in patients with ARDS, and successfully constructed the linkage between upregulated cellular MALAT1, downregulated exosomal miR-425, and PTEN protein level in vitro. These findings exhibit a possible role of MALAT1 during the pathophysiological processes of ARDS which needs to be further confirmed in vivo.

## Figures and Tables

**Table 1:** Clinical characteristics of ARDS patient population.

Phenotype	*n*	Age	Male/female	Pathogenic factors	Serum IL-1*β* (pg/ml)	Lung Ly6G + neutrophil (%)	PaO_2_/FiO_2_ (mm Hg)
Healthy controls	36	45.3	20/18	NA	11.1 ± 6.1	4.1 ± 0.9	427.8 ± 49.6
ARDS	36	46.7	22/14	Primary pneumonia	50.6 ± 21.9	11.1 ± 2.4	96.6 ± 42.8
	22	42.3	16/6	Trauma	43.6 ± 17.6	13.5 ± 2.8	150.2 ± 67.6
	7	44.2	4/3	Blood transfusion	42.5 ± 19.8	9.1 ± 4.7	195.9 ± 99.1

ARDS: acute respiratory distress syndrome; PaO_2_: partial pressure of arterial oxygen; FiO_2_: percentage of inspired oxygen; NA: not applicable. Values represent mean ± SD.

## Data Availability

The data used to support the findings of this study are available from the corresponding author upon request.

## Supplementary Materials

Supplementary Materials Fig. S1: miRNA level in the plasma exosomes. Total RNAs were extracted from plasmal exosome and candidate miRNA level was detected by qRT-PCR. Results were shown as box-whisker diagram, and data out of 95% CI are shown as dots. Results were analyzed by Student's *t*-test, and *p* < 0.05 was considered significant. Fig. S2: miR-425 level was not reduced by MALAT1 in the cells. A549 and HFL-1 cells were transfected with wildtype or mutant MALAT1 expression vector for 48 hours. miR-425 level was detected by qRT-PCR.
