# Supplementary figures and images for: Overexpression of MALAT1 Relates to Lung Injury through Sponging miR-425 and Promoting Cell Apoptosis during ARDS
Source: Can Respir J. 2019 Dec 1;2019:1871394. doi: 10.1155/2019/1871394 (PMC6913333; doi:10.1155/2019/1871394)

Figure S1

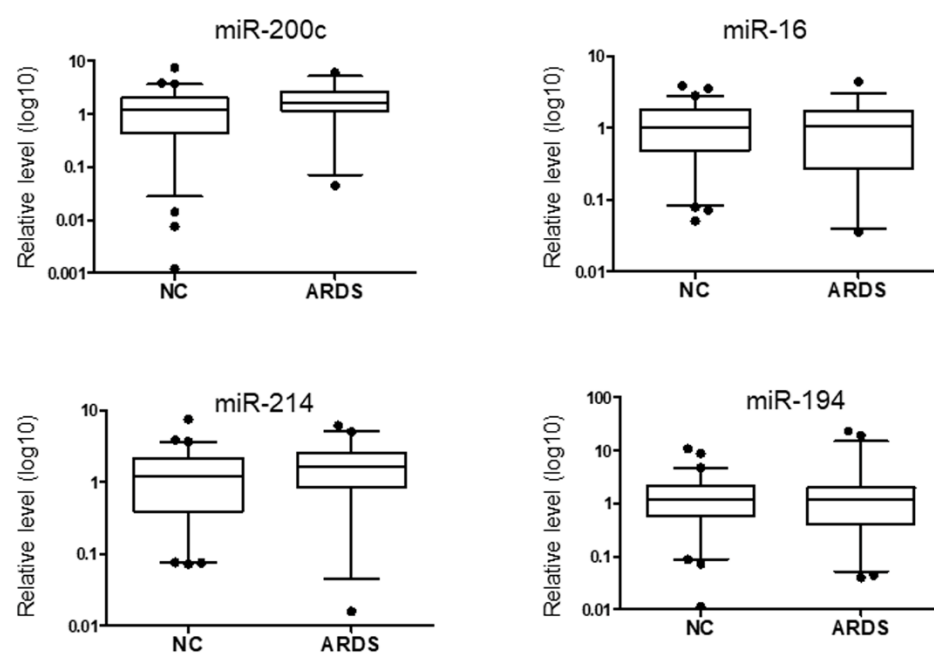

Fig.S1

Figure S2

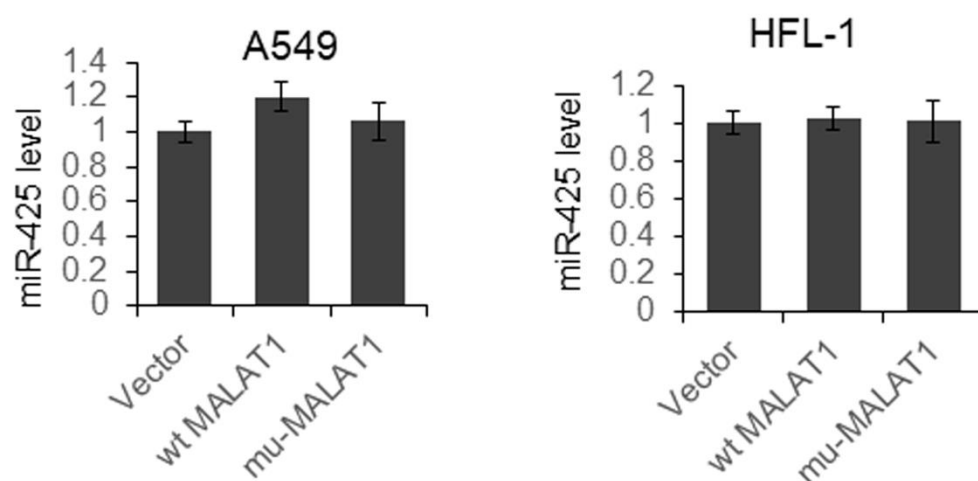

Fig.S2

Supplement: Supplementary Materials — Fig. S1: miRNA level in the plasma exosomes. Total RNAs were extracted from plasmal exosome and candidate miRNA level was detected by qRT-PCR. Results were shown as box-whisker diagram, and data out of 95% CI are shown as dots. Results were analyzed by Student's t-test, and p < 0.05 was considered significant. Fig. S2: miR-425 level was not reduced by MALAT1 in the cells. A549 and HFL-1 cells were transfected with wildtype or mutant MALAT1 expression vector for 48 hours. miR-425 level was detected by qRT-PCR. [file 1871394.f1.pdf]
